# On-Pump or Off-Pump Impact of Diabetic Patient Undergoing Coronary Artery Bypass Grafting 5-Year Clinical Outcomes

**DOI:** 10.31083/j.rcm2509349

**Published:** 2024-09-24

**Authors:** Fei Xu, Lei Li, Chenghui Zhou, Sheng Wang, Hushan Ao

**Affiliations:** ^1^Department of Anesthesiology, Beijing Anzhen Hospital, Capital Medical University, 100029 Beijing, China; ^2^Department of Cardiovascular Surgery, Affiliated Hospital of Weifang Medical University, 261071 Weifang, Shandong, China; ^3^Department of Anesthesiology, Cardiovascular Institute and Fuwai Hospital, Chinese Academy of Medical Sciences, 100037 Beijing, China

**Keywords:** coronary artery bypass graft, on-pump, off-pump, outcomes, diabetes

## Abstract

**Background:**

For diabetic patients undergoing coronary artery bypass grafting (CABG), there is still a debate about whether an off-pump or on-pump approach is advantageous.

**Methods:**

A retrospective review of 1269 consecutive diabetic patients undergoing isolated, primary CABG surgery from January 1, 2013 to December 31, 2015 was conducted. Among them, 614 received non-cardiopulmonary bypass treatment during their operation (off-pump group), and 655 received cardiopulmonary bypass treatment (on-pump group). The hospitalization outcomes were compared by multiple logistic regression models with patient characteristics and operative variables as independent variables. Kaplan-Meier curves and Cox proportional-hazard regression models for mid-term (2-year) and long-term (5-year) clinical survival analyses were used to determine the effect on survival after CABG surgery. In order to further verify the reliability of the results, propensity-score matching (PSM) was also performed between the two groups.

**Results:**

Five-year all-cause death rates were 4.23% off-pump vs. 5.95% on-pump (*p* = 0.044), and off-pump was associated with reduced postoperative stroke and atrial fibrillation.

**Conclusions:**

These findings suggest that off-pump procedures may have benefits for diabetic patients in CABG.

## 1. Introduction

Diabetes is a significant, well-established risk factor for cardiovascular disease [1]. As cardiovascular disease treatment strategies have been improved, the overall morbidity and mortality associated with cardiovascular events have declined [2]. Diabetic patients with acute cardiovascular events continue to have a poorer prognosis compared to those without diabetes [3].

Coronary artery bypass grafting (CABG) has become an effective method for coronary heart disease revascularization. Cardiopulmonary bypass (CPB) has been carried out since the 1960s. It has been widely used in cardiac surgery. In spite of this, CPB due to reperfusion injury, the release of inflammatory mediators, microthrombus formation and other causes of adverse effects on the body, also CABG perioperative period management adds new challenges [4].

Traditionally, CABG has been performed on-pump, which means using the CPB and cardioplegic arrest. CPB use has been associated with postoperative myocardial, pulmonary, renal and cerebral complications [5, 6]. Studies have shown that off-pump CABG may avoid many of these complications, leading to better clinical outcomes.

The comparative effectiveness of the off-pump and on-pump procedures have been debated but few studies have compared two different procedures of CABG in patients with diabetes.

Randomized trials have found that direct comparisons between off-pump and on-pump surgery in diabetic patients remain extremely limited. A 2017 meta-analysis conducted by Wang and colleagues concluded that no treatment-related differences were found in mortality, myocardial infarction (MI), or renal outcomes among diabetic patients [7].

In December 2016, the largest international multi-center, randomized, controlled clinical trial of off-pump vs. on-pump [CORONARY] reported clinical outcomes after a mean follow-up of 4.8 years of CABG. For patients with diabetes, the 5-year major adverse cardiac and cerebrovascular events (MACCE) outcomes of off-pump were lower than those of on-pump (22.7% vs. 26.1%) [8]. The off-pump techniques could offer the benefit of less inflammation and embolization with a reduction in atrial fibrillation with postoperative stroke.

In another study conducted one year later, no differences were seen in MACCE, repeat revascularization, and nonfatal myocardial infarction. However, the incidence of cardiac was worse with off-pump CABG than with on-pump CABG (9.0% vs. 6.25%) [9].

In light of this literature-based controversy, concerns have been raised about the disparate effects of off-pump surgery on long-term outcomes in patients with diabetes. We analyzed records of diabetic patients in China undergoing CABG surgery who were managed off-pump or on-pump to clarify the clinical effects.

## 2. Materials and Methods

This was a single-center, retrospective study of consecutive diabetic patients undergoing isolated primary CABG from January 1, 2013 to December 31, 2015. All subjects gave their written informed consent before they participated in the study.

The main focus was to compare off-pump with on-pump results in diabetic patients. The inclusion criteria of this study were: isolated CABG procedures. The exclusion criteria were: emergency surgery or re-operation. A total of 1269 met the inclusion criteria, of which 655 received the on-pump procedure and 614 received the off-pump procedure. Diabetes was defined as patients who were treated for diabetes with either medication or lifestyle changes at baseline or those patients with at least 2 fasting blood glucose measurements >126 mg/dL but were not treated with medication or lifestyle changes. In-hospital, mid-term, and 5-year survival were studied.

### 2.1 Surgical Techniques 

Anesthesia and surgical techniques were standardized for all patients. Patients were operated on through the mid-sternotomy. All operations were performed by experienced surgeons. The decision to perform off-pump or on-pump surgery was based on clinical field practice patterns, individual surgeon preference, the patient’s clinical characteristics and the perceived quality of the target vessel. After surgery, patients were transferred to the intensive care unit (ICU). Once the following criteria were met: normal body temperature, consciousness, hemodynamic stability, and no significant bleeding, the patients were extubated.

### 2.2 Study Outcomes 

All results were specified before analysis and were defined by the protocol. The primary outcome of this study was postoperative in-hospital, mid-term (2-year), and long-term (5-year) survival after a clinical CABG surgery procedure. In-hospital mortality was defined as death during the primary hospitalization. Myocardial infarction was defined as the appearance of new Q waves in two or more consecutive leads on the electrocardiogram (ECG). Cerebrovascular accidents were defined as a loss of central neurologic function lasting more than 72 hours. Renal failure was defined as the need for dialysis to treat chronic oliguria or anuria; stroke as a central neurological deficit lasting more than 72 hours; coma as being unresponsive for more than 24 hours; encephalopathy as being a reversible neurological deficit (recovery within 72 h of onset). Survival outcomes were recorded from 2-year and 5-year follow-ups. The secondary outcomes included the incidence of postoperative MI, stroke, new-onset atrial fibrillation, renal failure, and repeated revascularization. As well as the incidence of stroke and MI 2 years and 5 years post-procedure.

### 2.3 Statistical Analysis 

Continuous variables were expressed as mean ± standard deviation, and comparison was performed by using the *t* test. Categorical variables were described as frequency and percentages, and comparison was performed by the chi-square test. The hospitalization outcomes were compared by multiple logistic regression models with patient characteristics and operative variables as independent variables and odds ratios (ORs) were estimated. Kaplan-Meier curves and Cox proportional-hazard regression models for mid-term (2-year) and long-term (5-year) survival analyses were used to determine the effect on survival after CABG surgery. Hazard ratios (HRs) were estimated by Cox proportional-hazard regression models. Confounding factors were included in the models if *p* ≤ 0.05.

For adjusting between-group differences, propensity-score matching (PSM) was also performed, where 504 patients undergoing an off-pump procedure were matched in a 1:1 ratio to patients receiving an on-pump procedure.

The statistical tests were analyzed by the SAS 9.13 software (SAS Institute, Cary, NC, USA).

## 3. Results

**Fig. 1.**
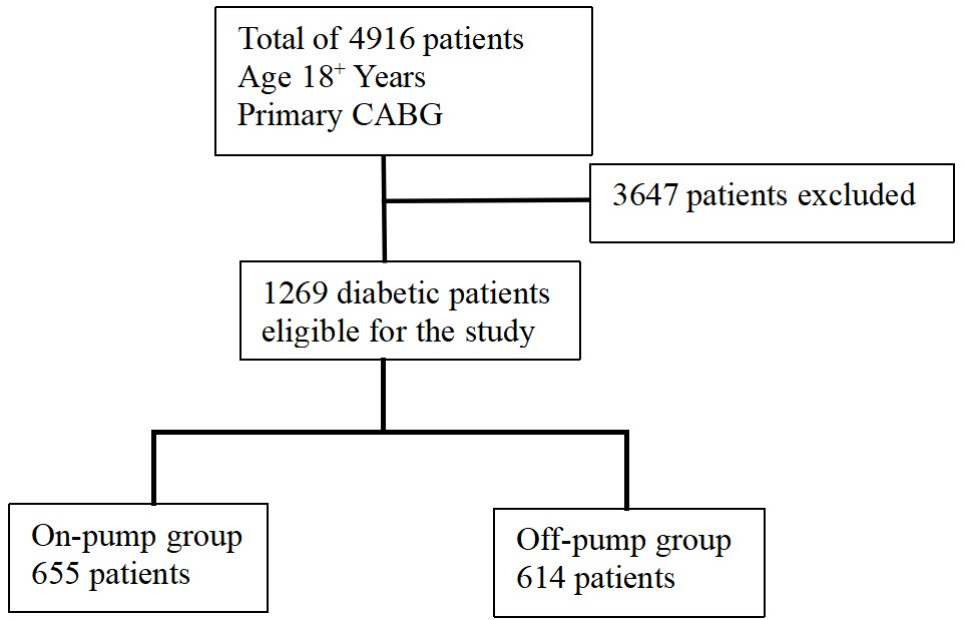
**Study population recruitment summary**. CABG, coronary artery bypass grafting.

Fig. 1 shows the study population recruitment summary. A total of 1269 patients were ultimately included in the study. Among them, 614 received non-cardiopulmonary bypass treatment during their operation (off-pump group), and 655 received cardiopulmonary bypass treatment (on-pump group).

### 3.1 Baseline characteristics 

The demographic and clinical data of the patients are shown in Table 1. There were no significant differences between the two groups in terms of history of body mass index (BMI), sex, smoking, family medical history, hypertension, renal failure, cerebrovascular events, MI, atrial fibrillation, preoperative creatinine, left ventricular ejection fraction (LVEF), number of anastomosis and rate of complete revascularization. However, the off-pump group patients were older (61.52 ± 8.68 vs. 60.47 ± 7.45), and a higher percentage had peripheral artery disease (16.3% vs. 5.5%), and thrombolytic therapy (10.1% vs. 5.2%). Patients in the on-pump group were more likely to develop left main coronary disease (25.9% vs. 31.0%), heart failure (1.3% vs. 3.7%) and diseased coronary artery (2.80 ± 0.49 vs. 2.87 ± 0.35).

**Table 1.** **Demographic and clinical characteristics**.

Variable	Entire cohort		*p* value
	Off-pump CABG	On-pump CABG	
	N = 614	N = 655	
Age (yrs.)	61.52 ± 8.68	60.47 ± 7.45	0.022
BMI (kg/m^2^)	25.59 ± 3.15	26.82 ± 16.37	0.060
Female	117 (19.1%)	138 (21.1%)	0.371
Smoking	300 (48.9%)	323 (49.3%)	0.872
Family history	40 (6.5%)	55 (8.4%)	0.203
Hypertension	425 (69.2%)	441 (67.3)	0.470
Hyperlipidemia	231 (37.6%)	287 (43.8)	0.025
History of renal failure	9 (1.5%)	3 (0.5%)	0.064
Creatinine (µmol/L)	88.09 ± 22.87	87.87 ± 24.52	0.869
Cerebrovascular events	43 (7.0%)	51 (7.8%)	0.595
Peripheral artery disease	100 (16.3%)	36 (5.5%)	<0.001
Thrombolytic therapy	62 (10.1%)	34 (5.2%)	0.001
Myocardial infarction	291 (47.4%)	341 (52.1%)	0.097
Diseased coronary artery	2.80 ± 0.49	2.87 ± 0.35	0.002
Left main disease	159 (25.9%)	203 (31.0%)	0.044
Heart failure	8 (1.3%)	24 (3.7%)	0.007
Atrial fibrillation	17 (2.8%)	19 (2.9%)	0.887
LVEF	58.85 ± 8.93	58.37 ± 9.97	0.364
Number of anastomosis	2.62 ± 0.98	2.47 ± 0.68	0.157
Rate of complete revascularization	491 (80.0%)	530 (80.9%)	0.371
Steletonized internal mammary artery	296 (48.2%)	337 (51.5%)	0.248

CABG, coronary artery bypass grafting; LVEF, left ventricular ejection fraction; BMI, body mass index.

### 3.2 Postoperative Outcomes

Table 2 illustrates postoperative clinical outcomes between two groups. In-hospital mortality was 1.26% for the entire cohort, 1.14% for the off-pump CABG group, and 1.37% for the on-pump CABG group. There were no differences in in-hospital mortality between the two groups (adjusted OR 1.145, 95% confidence interval [CI]: 0.056 to 3.737). However, stroke had more incidence rate in the on-pump CABG group (adjusted OR 7.892, 95% CI: 1.698 to 12.727) and new-onset atrial fibrillation had more incidence rate in the on-pump group (adjusted OR 1.427, 95% CI: 1.137 to 2.191).

**Table 2.** **Postoperative outcomes with multiple logistic-regression analysis**.

Variable	Entire cohort		Adjusted OR	95% CI	*p* value
	Off-pump CABG	On-pump CABG			
	N = 614	N = 655			
Stroke	2 (0.326)	12 (1.83)	7.892	1.698–12.727	0.008
Renal failure	3 (0.489)	7 (1.07)	2.224	0.548–9.021	0.263
Atrial fibrillation	48 (7.82)	71 (10.8)	1.427	1.137–2.191	0.041
Mortality	7 (1.14)	9 (1.37)	1.145	0.056–3.737	0.467
Myocardial infarction	3 (0.489)	9 (1.37)	1.889	0.440–8.107	0.392
Repeated revascularization	7 (1.14)	10 (1.53)	1.174	0.438–3.144	0.750

CABG, coronary artery bypass grafting; OR, odds ratio.

### 3.3 Follow-up Outcomes

After 2 years of follow-up, 38 of the 1269 patients (2.99%) had died from all causes. 2-year mortality was analyzed by Cox proportional-hazard regression models between the two groups (2.44% vs. 3.51%; adjusted HR, 1.145; 95% CI 0.826 to 3.175, *p* = 0.591). Also, there were no significant differences in 2-year MI and stroke between the two groups (Table 3).

**Table 3.** **2-year outcomes with Cox proportional-hazard models**.

Variable	Entire cohort		Adjusted HR	95% CI	*p* value
	Off-pump CABG	On-pump CABG			
	N = 614	N = 655			
Mortality	15 (2.44%)	23 (3.51%)	1.145	0.826–3.175	0.591
MI	3 (0.489%)	5 (0.763%)	1.228	0.072–5.274	0.677
Stroke	25 (4.07%)	37 (5.65%)	2.354	0.468–2.137	0.365

CABG, coronary artery bypass grafting; MI, myocardial infarction; HR, hazard ratio.

After 5 years of follow-up, 65 of the 1269 patients (5.12%) had died from all causes. 5-year mortality was analyzed for significant differences between the two groups (4.23% vs. 5.95%; adjusted HR, 1.634; 95% CI 1.154 to 2.800, *p* = 0.044) by Cox proportional-hazard regression models. However, there were no significant differences in 5-year MI and stroke between the two groups (Table 4).

**Table 4.** **5-year outcomes with Cox proportional-hazard models**.

Variable	Entire cohort		Adjusted HR	95% CI	*p* value
	Off-pump CABG	On-pump CABG			
	N = 614	N = 655			
Mortality	26 (4.23%)	39 (5.95%)	1.634	1.154–2.800	0.044
MI	10 (1.63%)	11 (1.68%)	0.861	0.340–2.182	0.752
Stroke	80 (13.03%)	106 (16.18%)	0.885	0.650–1.204	0.437

CABG, coronary artery bypass grafting; MI, myocardial infarction; HR, hazard ratio.

**Fig. 2.**
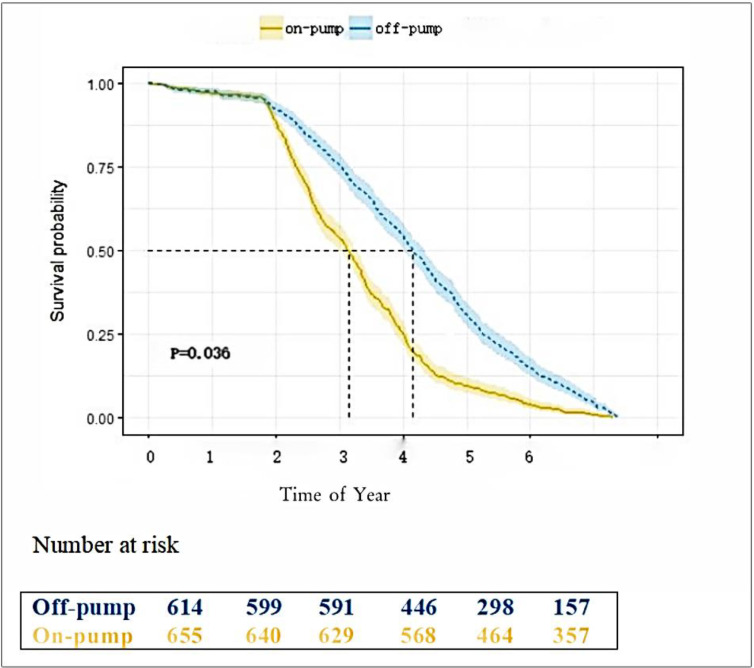
**5-year survival rate after surgery**.

Fig. 2 shows the Kaplan-Meier event-free survival analysis of 5-year mortality after surgery.

### 3.4 Propensity-Matched Groups and Outcomes

Following adjustment by PSM, the two derived groups included 504 patients with well-matched and balanced baseline characteristics (Table 5). Postoperative stroke and new-onset atrial fibrillation had reduced incidence rates in the on-pump CABG group, which confirmed the result of multi-variable logistic regression analysis (Table 6). Further comparing the two well-matched groups, there was no significant difference in 2-year survival (Table 7) but there was reduced mortality in the off-pump group at 5 years post-treatment (Table 8) as described in the analysis above.

**Table 5.** **Demographic and clinical characteristics for propensity score match**.

Variable	Propensity score match		*p* value	SMD
	Off-pump CABG	On-pump CABG		
	N = 504	N = 504		
Age (yrs.)	61.39 ± 8.62	60.90 ± 7.50	0.336	–0.063
BMI (kg/m^2^)	25.39 ± 3.21	25.40 ± 2.88	0.962	0.071
Female	102 (20.2%)	99 (19.6%)	0.813	–0.065
Smoking	237 (47.0%)	241 (47.8)	0.801	0.057
Family history	34 (6.7%)	35 (6.9%)	0.901	0.077
Hypertension	338 (67.1%)	340 (67.5%)	0.893	0.053
Hyperlipidemia	196 (38.9%)	195 (38.7%)	0.948	0.044
History of renal failure	6 (1.2%)	3 (0.6%)	0.315	0.081
Creatinine (µmol/L)	87.96 ± 22.45	87.40 ± 25.11	0.710	–0.072
Cerebrovascular events	41 (8.1%)	36 (7.1%)	0.553	–0.086
Peripheral artery disease	38 (7.5%)	35 (6.9%)	0.715	–0.058
Thrombolytic therapy	34 (6.7%)	32 (6.3%)	0.799	–0.053
Myocardial infarction	242 (48.0%)	238 (47.2%)	0.801	–0.049
Diseased coronary artery	2.86 ± 0.36	2.85 ± 0.38	0.735	–0.026
Left main disease	139 (27.6%)	140 (27.8%)	0.944	0.035
Heart failure	8 (1.6%)	6 (1.2%)	0.590	–0.072
Atrial fibrillation	13 (2.6%)	12 (2.4%)	0.840	–0.039
LVEF	58.93 ± 8,91	58.56 ± 9.81	0.536	–0.087
Number of anastomosis	2.61 ± 0.57	2.57 ± 0.65	0.573	–0.029
Rate of complete revascularization	405 (80.4%)	402 (79.8%)	0.812	–0.034
Steletonized internal mammary artery	282 (56.0%)	305 (60.5%)	0.142	0.056

CABG, coronary artery bypass grafting; SMD, standardized mean difference; LVEF, left ventricular ejection fraction; BMI, body mass index.

**Table 6.** **Postoperative outcomes with propensity score match**.

Variable	Propensity score match		*p* value
	Off-pump CABG	On-pump CABG	
	N = 504	N = 504	
Stroke	0	7 (1.4%)	0.008
Renal failure	2 (0.4%)	3 (0.6%)	0.654
Atrial fibrillation	35 (6.9%)	59 (11.7%)	0.009
Mortality	6 (1.2%)	5 (1.0%)	0.413
Myocardial infarction	3 (0.6%)	6 (1.2%)	0.315
Repeated revascularization	7 (1.4%)	7 (1.4%)	1.000

CABG, coronary artery bypass grafting.

**Table 7.** **2-year outcomes with propensity score match**.

Variable	Propensity score match		*p* value
	Off-pump CABG	On-pump CABG	
	N = 504	N = 504	
Mortality	12 (2.38%)	19 (3.77%)	0.202
MI	2 (0.397%)	5 (0.992%)	0.256
Stroke	19 (3.77%)	26 (5.16%)	0.286

CABG, coronary artery bypass grafting; MI, myocardial infarction.

**Table 8.** **5-year outcomes with propensity score match**.

Variable	Propensity score match		*p* value
	Off-pump CABG	On-pump CABG	
	N = 504	N = 504	
Mortality	21 (4.2%)	36 (7.1%)	0.041
MI	6 (1.2%)	8 (1.6%)	0.590
Stroke	58 (11.5%)	79 (15.7%)	0.054

CABG, coronary artery bypass grafting; MI, myocardial infarction.

## 4. Discussion

At present, the prevalence of type 2 diabetes worldwide is increasing year by year [10], and most patients with coronary heart disease have abnormal glucose metabolism [11]. Diabetic patients are prone to diffuse and rapidly progressive atherosclerosis. Therefore, many diabetic patients with coronary heart disease have polyangiopathy or severe narrowing of the blood vessels. This not only increases the risk of revascularization, but also increases the risk of a poor prognosis after surgery or percutaneous revascularization [12]. In addition, patients with diabetes are one of the most important subgroups of patients with a high risk of disease progression and complications after coronary artery bypass grafting [13].

The main finding of this study was that in patients with diabetes, off-cardiopulmonary bypass surgery was related to a lower risk of postoperative stroke and new atrial fibrillation compared to cardiopulmonary bypass. The 5-year mortality rate was significantly reduced. Our results are consistent with previous reports on the effect of off-pump surgery on beneficial outcomes in type 2 diabetes patients [14, 15].

An international multi-center randomized controlled clinical trial [CORONARY] reported clinical outcomes after off-cardiopulmonary bypass compared to cardiopulmonary bypass at 5 years follow-up. For diabetic patients, the 5-year major adverse cardiovascular event (MACE) outcome in the off-cardiopulmonary bypass CABG group was lower than that in the cardiopulmonary bypass CABG group [14]. In another study, Off-pump as shown to have a lower incidence of postoperative neurological complications in diabetic patients compared with on-pump group [16]. Our results showed that the average stroke rate of patients with diabetes who underwent cardiopulmonary bypass increased by 5.9 times, which is the independent risk factor for neurological complications [17]. Cardiopulmonary bypass and aortic manipulation may lead to cerebral embolism, which is an important potential mechanism for postoperative stroke.

None of these studies reported a benefit for new cases of atrial fibrillation after surgery. This study is the first to show that the incidence of new atrial fibrillation after surgery is lower in patients with type 2 diabetes who receive off-pump CABG. Atrial fibrillation is a common complication after coronary artery bypass transplantation [18]. After CPB, patients with diabetes have an increased incidence of atrial fibrillation after CABG, which is due to the systemic inflammatory response syndrome caused by CPB. The release of stress hormones in the body leads to a rapid rise in blood sugar, which makes diabetic patients unable to carry out normal glucose metabolism. Further, this can also induce ischemia reperfusion injury [19].

Compared with on-pump CABG, the use of off-pump CABG has been controversial with obvious advantages and disadvantages. In this study, we found that the rates of 5-year mortality, in-hospital stroke, and new-onset atrial fibrillation were reduced after off-pump CABG in diabetic patients. The patterns suggest these trends would be likely to increase over the long term. Our finding suggests that off-pump CABG could be used for patients with diabetes for increased benefit compared to on-pump CABG.

One limitation is that it was not randomized between the two surgical methods. Therefore, we used two statistical methods to compare the off-pump group with the on-pump group, allowing us to make reliable inferences. The estimates obtained from multi-variable regression models may not effectively account for treatment selection bias, but represented real world situations in which physicians were allowed to choose between off-pump and on-pump CABG as the preferred method for providing surgical revascularization. On the other hand, the estimates obtained based on a matched subset of patients ensured that the clinical covariates were evenly distributed between the two groups, thus to estimate the effect of off-pump in patients undergoing CABG procedures actually.

Considering the technical difficulties of using the right internal thoracic artery and the higher incidence of sternal deep wound infections, patients undergoing CABG received it only to the left internal thoracic artery and not the bilateral internal thoracic artery in our hospital. So, the role of bilateral internal mammary artery (BIMA) vs single internal mammary artery (SIMA) in diabetic patients could not be evaluated.

## 5. Conclusions

Using an off-pump procedure may have more benefits for diabetic patients undergoing CABG compared to using an on-pump procedure.

## Availability of Data and Materials

The data sets generated and/or analyzed during the current study are not publicly available but are available from the corresponding author on reasonable request.
